# Quasistatic Cavity Resonance for Ubiquitous Wireless Power Transfer

**DOI:** 10.1371/journal.pone.0169045

**Published:** 2017-02-15

**Authors:** Matthew J. Chabalko, Mohsen Shahmohammadi, Alanson P. Sample

**Affiliations:** Disney Research, Pittsburgh, 4720 Forbes Avenue, Lower Level, Suite 110, Pittsburgh, PA 15213 United States of America; West Virginia University, UNITED STATES

## Abstract

Wireless power delivery has the potential to seamlessly power our electrical devices as easily as data is transmitted through the air. However, existing solutions are limited to near contact distances and do not provide the geometric freedom to enable automatic and un-aided charging. We introduce quasistatic cavity resonance (QSCR), which can enable purpose-built structures, such as cabinets, rooms, and warehouses, to generate quasistatic magnetic fields that safely deliver kilowatts of power to mobile receivers contained nearly anywhere within. A theoretical model of a quasistatic cavity resonator is derived, and field distributions along with power transfer efficiency are validated against measured results. An experimental demonstration shows that a 54 m^3^ QSCR room can deliver power to small coil receivers in nearly any position with 40% to 95% efficiency. Finally, a detailed safety analysis shows that up to 1900 watts can be transmitted to a coil receiver enabling safe and ubiquitous wireless power.

## Introduction

Advances in wireless communication technologies such as WiFi have led to the ubiquitous deployment of hotspots allowing users to seamlessly connect their mobile devices simply by entering their home or office. In contrast, wireless power delivery has not benefited from the same advances in technology. Applications in robotics [1, 2], medical implants [3, 4], and consumer electronics [5, 6] are still limited to near contact transfer distances and thus do not provide the geometric freedom and ease of use the term “wireless” suggests.

However, this has not always been the case. At the turn of the 1900s Nikola Tesla routinely demonstrated room wide wireless power transfer using what is commonly referred to as a Tesla coil [7]. While this early foray into wireless power attempted to maximize mobility and ease of power distribution, at the time the hazardous effects of prolonged exposure to large electric fields was unknown. Regulatory agencies have since adopted strict safety guidelines to ensure the health and safety of the general public [8–10].

This has resulted in a long-standing tradeoff between the range at which a device can be wirelessly powered and the maximum amount of power that can be safely delivered. For example, radiative transfer methods have tightly coupled electric and magnetic fields that propagate over long distances and are typically used for radio communication. These far-field wireless power techniques [11, 12] have not found wide spread use, since they are limited to delivering only a few milliwatts of power due to health and safety concerns. In contrast, non-radiative transfer systems such as inductive charging cradles [13, 14] and resonant charging pads [15, 16] can safely deliver 10s-100s of watts of power by loosely decoupling the magnetic fields–which are used to transfer power–from the potentially harmful electric fields [17]. However, near-field coupling is a highly localized phenomenon and transfer efficiency drops off rapidly as the source and receiver are separated by more than a coil diameter [18, 19]. Likewise, it is not possible to strongly couple coils of drastically different sizes [20].

Drawing upon recent work using far-field standing electromagnetic waves to generate uniform field patterns in a metallic chamber [21, 22], we introduce quasistatic cavity resonance (QSCR); which can be used to create near-field standing waves that fill the interior of the resonant structure with uniform magnetic fields, allowing for strong coupling to small receivers contained within. This is accomplished by stimulating the resonant electromagnetic mode of a specially designed, enclosed metallic structure such that induced currents flowing through the walls, ceiling and floor are channeled through discrete capacitors. These oscillating currents in turn generate magnetic fields that permeate the interior of the structure, thus enabling wireless power transfer to receivers contained within, while simultaneously isolating the potentially harmful electric fields in capacitors. This high Q-factor structure efficiently stores electromagnetic energy, and the discrete capacitors allow the resonant frequency to be lowered to a point where the cavity enters the deep sub-wavelength regime, effectively separating the magnetic field from the electric field.

**Fig 1:**
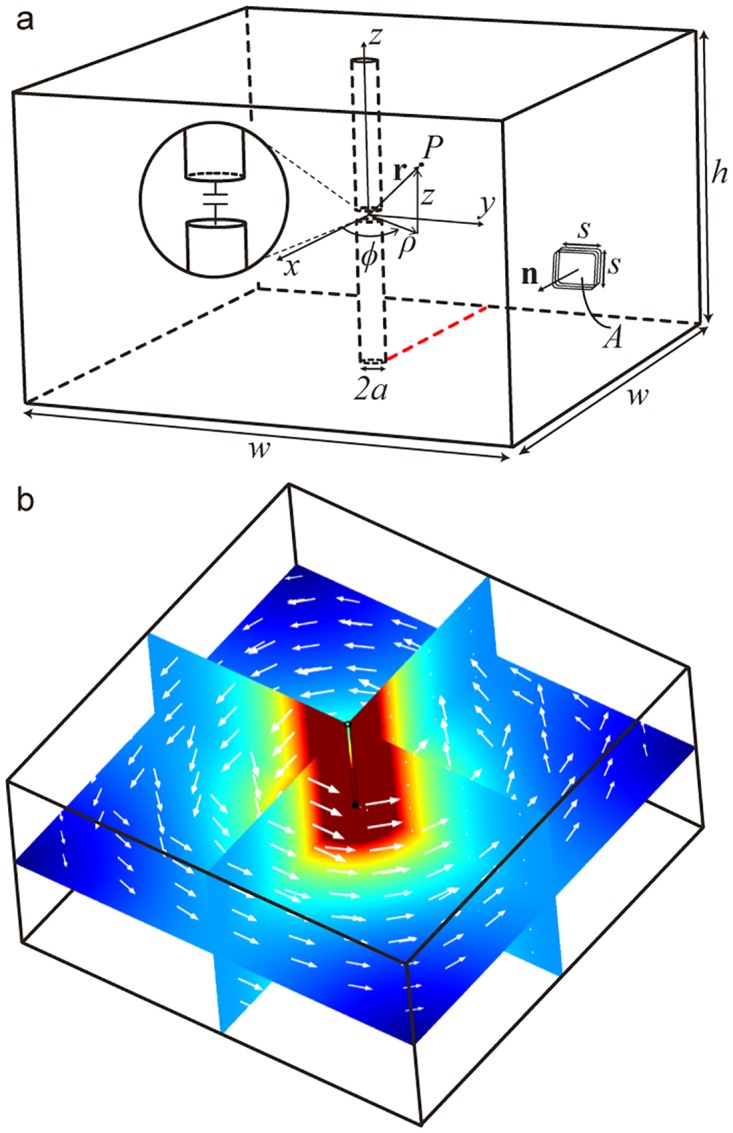
Canonical example of a quasistatic cavity resonator. (a) Geometric setup along with reference geometry and coordinate systems. A square coil receiver is depicted within the QSCR. (b) Magnetic field, H. Color is magnitude (red, large; blue, small); Arrows are magnetic field vectors.

A conceptual diagram of a QSCR is shown in Fig 1a, which depicts a generic rectangular cavity with a central pole that incorporates the capacitors. The magnetic fields (shown in Fig 1b) are highly uniform and decay at a rate of less than 1/*ρ* towards the walls, making it possible to strongly couple to coil receivers 1000s of times smaller than the size of the QSCR. Furthermore, deep sub-wavelength operation results in a magnetic field to electric field ratio that is on average 100 times greater than in free space, allowing for a substantially higher level of power to be safely transferred. By scaling the quasistatic cavity resonator up to the size of a living room, office, or warehouse it is possible to deliver safe and ubiquitous wireless power to small mobile devices contained nearly anywhere within. While QSCR enabled spaces do require purpose-built structures, as the walls must be conductive, it offers a substantial improvement in the tradeoff between range and the magnitude of power that can be safely delivered. Since coupled resonators only share energy efficiently with objects of the same resonant frequency, interactions with common everyday objects and materials is minimal, allowing for typical home and office furnishing to be included in the chamber. Ultimately this unexplored form of wireless power offers a seamless charging experience where a user’s device can be charged when entering a QSCR enabled space as easily as data is transfer through the air.

## Theory

While QSCRs can take many forms, the topology of a capacitively loaded vertical pole position in the center of rectangular cavity resonator was chosen due to its ease of construction and analysis. The goal of the following derivation is to determining the power transfer efficiency to a small coil receiver located inside this QSCR enable space. Our analysis begins by deriving the fields generated by the current flowing through the pole, which is approximated by a line current at *ρ* = 0. Fundamentally, the electromagnetic fields due to any arbitrary current source can be derived using 3 general steps. First, the magnetic vector potential **A** is determined (where bold type indicates vector quantities). Next, the magnetic field distribution is obtained using the definition **H** = 1/*μ*_*o*_∇ × **A**. Lastly, the electric field is recovered using Ampere’s law. The reference geometry and coordinate systems used in the following derivation are shown in Fig 1a. The general expression for **A** [23, 24] in steady state due to a current distribution, **J**, is

$$\begin{array}{c} \text{A}\left(\text{r}\right)=\frac{\mu_{o}}{4\pi }\int \frac{\text{J}\left({\text{r}}^{\prime }\right)e^{-jk|\text{r}-{\text{r}}^{\prime }|}}{|\text{r}-{\text{r}}^{\prime }|}{\text{dr}}^{\prime 3} \end{array}$$ (1)

where *μ*_*o*_ is the permeability of free space, *j* is the imaginary unit, **r** is the vector from the origin to the field observation point, *P*, and **r**′ is the vector from the origin to the current source. Additionally, *k* is the wavenumber. To evaluate Eq (1) the current distribution along the pole, **J**(**r**′), is needed:

$$\begin{array}{c} \text{J}\left({\text{r}}^{\prime }\right)=I_{o}\cos^{2}\left[k\left(\frac{h}{2}-|z|\right)\right]\delta \left(\rho \right)\;{\text{a}}_{\text{z}} \end{array}$$ (2)

Here, *I*_*o*_ is the peak current, *h* is the height of the pole, and *δ*(*ρ*) is the Dirac delta function, which enforces that the current source is a line centered at *ρ* = 0. This is a reasonable approximation when the radius of the pole, *a*, is small compared to the size of the room. Additionally, since the frequency of operation results in a free space wavelength that is much longer then the QSCR dimensions (i.e. the system is in the deep sub-wavelength region), *k*|**r** − **r**′| will be small and the exponential in Eq (1) can be approximated by unity. Thus, substitution of Eq (2) into Eq (1) then yields

$$\begin{array}{c} \text{A}=\frac{\mu_{o}I_{o}}{4\pi }\int_{-\infty }^{\infty }\frac{\cos^{2}\left[k\left(\frac{h}{2}-|z^{\prime }|\right)\right]}{{\left[\rho^{2}+{\left(z-z^{\prime }\right)}^{2}\right]}^{\frac{1}{2}}}dz^{\prime }\;{\text{a}}_{\text{z}} \end{array}$$ (3)

In the above, the current source is taken to be infinitely long, which is a reasonable assumption since the floor and ceiling are conductive resulting in *z*-directed virtual current sources via image theory. The integral in Eq (3) has no closed form solution and approximate equations for the H and E fields due to current in the QSCR’s central pole alone can be derived using a few terms in a Taylor series expansion, the details of which can be found in the supplementary file, S1 Appendix.

$$\begin{array}{c} \text{H}=H_{\phi }{\text{a}}_{\phi }\approx \frac{I_{o}}{2\pi }\frac{\cos^{2}\left[k\left(\frac{h}{2}-|z|\right)\right]}{\rho }{\text{a}}_{\phi } \end{array}$$ (4)

$$\begin{array}{c} \text{E}\approx \frac{-j\eta_{o}I_{o}}{2\pi }\sin\left[k\left(2|z|-h\right)\right][\frac{z^{3}}{{\left(\rho^{2}+z^{2}\right)}^{3/2}\rho }{\text{a}}_{\rho }+\frac{\rho^{2}+2z^{2}}{{\left(\rho^{2}+z^{2}\right)}^{3/2}}{\text{a}}_{\text{z}}] \end{array}$$ (5)

where *η*_*o*_ is the impedance of free space.

**Fig 2:**
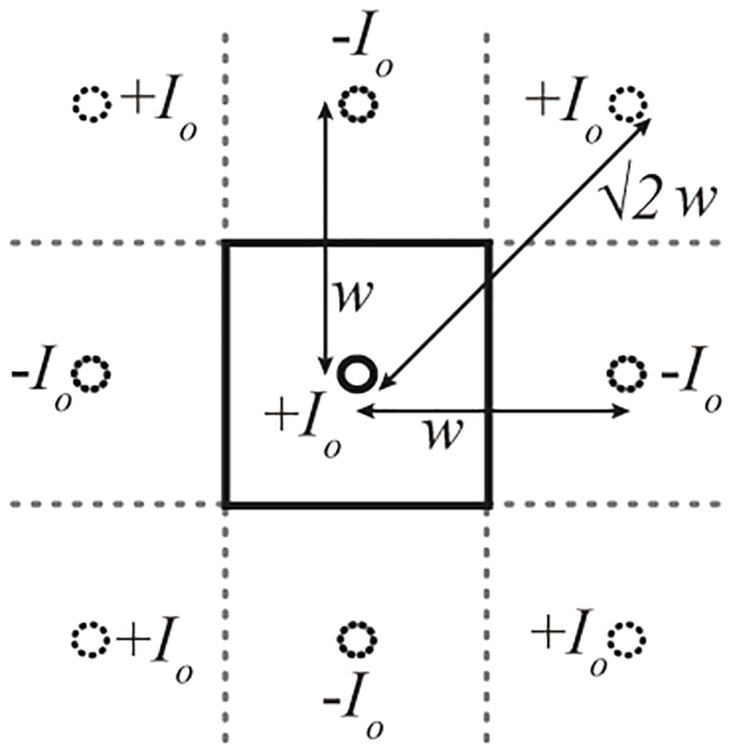
Top view of the QSCR. Locations and polarity of image currents due to the conducting walls (dotted circles). Solid circle in the center is the real pole current, and the black square is the QSCR outline from above.

Finally, to be complete about the total electric and magnetic fields, the effect of the walls needs to be taken into account. This can be handled by taking the expressions above for the electric and magnetic fields and then invoking classical image theory for a line current above a series of infinite ground planes. In this way, the total E or H fields within the cavity are the sum of the fields due to the pole itself, plus 8 images due to the walls. The contributions to the fields from a given image current are obtained by taking the field solutions for a pole centered at (0,0,0) and shifting the *x* and *y* coordinates to the location of the image, shown as dotted lines in Fig 2. Mathematically, the summation of the fields due to all images plus those due to the original line current transforms Eqs (4) and (5) to

$$\begin{array}{c} \text{H}=\frac{1}{2\pi }\sum_{m=-1}^{1}\sum_{l=-1}^{1}\frac{-I_{o}{\left(-1\right)}^{l+m}\left(y-mw\right)}{{\left(x-lw\right)}^{2}+{\left(y-mw\right)}^{2}}{\text{a}}_{\text{x}}+\frac{I_{o}{\left(-1\right)}^{l+m}\left(x-lw\right)}{{\left(x-lw\right)}^{2}+{\left(y-mw\right)}^{2}}{\text{a}}_{\text{y}} \end{array}$$ (6)

$$\begin{array}{ccc} \text{E} & = & A\sum_{m=-1}^{1}\sum_{l=-1}^{1}I_{o}{\left(-1\right)}^{l+m}\{\frac{z^{3}u}{{\left[u^{2}+v^{2}+z^{2}\right]}^{3/2}[u^{2}+v^{2}]}{\text{a}}_{\text{x}}\\ & & +\frac{z^{3}v}{{\left[u^{2}+v^{2}+z^{2}\right]}^{3/2}[u^{2}+v^{2}]}{\text{a}}_{\text{y}}+\frac{u^{2}+v^{2}+2z^{2}}{{\left[u^{2}+v^{2}+z^{2}\right]}^{3/2}}{\text{a}}_{\text{z}}\} \end{array}$$ (7)

where *u* = (*x* − *lw*), *v* = (*y* − *mw*), and *A* = −*jη*_*o*_ sin[*k*(2|*z*| − *h*)]/(2*π*).

In the above, the length and width of the QSCR are both *w*. Note that the cos^2^ term originally present in Eq (4) has been dropped since it is very close to unity, regardless of the observation point’s *z*-height. The above two expressions give the *total* magnetic and electric fields at any point within the cavity, and are valid for a general quasistatic cavity resonator with the topology of Fig 1.

Having derived expressions for the electric and magnetic fields we turn our attention to predicting coupling to a coil receiver and WPT efficiency. It is known from coupled mode theory [18, 21] that the three elements necessary to determine system efficiency are the quality factor of the transmitter, the quality factor of the receiver, as well as the coupling coefficient between the two [21, 22]. In this case, the coupling coefficient, *κ* (rad/s) between the cavity resonator and small receivers inside the resonator are defined using the following expressions:

$$\begin{array}{c} \kappa =\frac{\sqrt{2}}{4}\frac{\omega_{1}\beta }{\sqrt{L_{2}\alpha }} \end{array}$$ (8)

$$\begin{array}{c} \alpha =\underset{V}{\;\int \int \int \;}\frac{\mu_{o}}{2}{\left|\text{H}\right|}^{2}\;dV \end{array}$$ (9)

$$\begin{array}{c} \beta =\underset{A}{\;\int \int \;}\mu_{o}\text{H}\cdot \text{n}\;dA \end{array}$$ (10)

The expression for *α* can be recognized as the total magnetic energy stored in the room; similarly, *β* is the flux captured by a closed loop receiver. In the above, *V* is the volume of the enclosed cavity, **n** is the unit normal vector of the closed loop receiver’s surface, *ω*_1_ is the resonant frequency of the QSCR, *A* is the area enclosed by the receiver, and *L*_2_ is the receiver’s inductance. Evaluation of *α* and *β*, followed by substitution into Eq (8) produces the coupling coefficient between the QSCR and a receiver.

Once the coupling coefficient is known, the expected WPT efficiency (*G*_*max*_) can be predicted assuming a perfectly lossless bi-conjugate impedance match [22, 25, 26]. While this is an upper bound on WPT efficiency, implementations of discrete matching networks can achieve very similar performance in practice [26, 27]. Knowing only *κ*, the Q-factor of the chamber (*Q*_1_), and Q-factor of the receiver (*Q*_2_), *G*_*max*_ can be computed from the following expressions [22]:

$$\begin{array}{ccc} G_{max} & = & \frac{\chi }{{\left(1+\sqrt{1+\chi }\right)}^{2}}\\ \chi & = & \frac{4Q_{1}Q_{2}{\left|\kappa \right|}^{2}}{\omega_{1}\omega_{2}} \end{array}$$ (11)

Before proceeding to experimental results, a few final comments on Eqs (10) and (9) are helpful to extend their utility. First, we mention that the computation of *β* in Eq (10) is more straightforward for square shaped coils and so influenced our design of an experimental receiver, to be presented in later sections.

Next, notice that once *α* is computed, the effective inductance looking into the gap in the pole can be determined by using the definition of the magnetic energy (*w*_*m*_) stored in an inductor, $w_{m}=1/2L_{1}I_{o}^{2}$. Without loss of generality, we can assume *I*_*o*_ = 1; then, since *w*_*m*_ = *α*, the result for the inductance of the cavity is

$$\begin{array}{c} L_{1}=2\alpha \end{array}$$ (12)

Given this inductance, the capacitance, *C*_*o*_, that needs to be inserted across the pole’s gap to produce resonance at a given frequency, *f*_1_, can be computed from the well-known expression for the resonance of an *LC* resonator, $\omega_{1}=2\pi f_{1}=1/\sqrt{L_{1}C_{1}}$, yielding:

$$\begin{array}{c} C_{1}=\frac{1}{{\left(2\pi f_{1}\right)}^{2}2\alpha } \end{array}$$ (13)

Both Eqs (12) and (13) are fundamental design equations required for designing the QSCR presented in this article.

## Experimental Results

**Fig 3:**
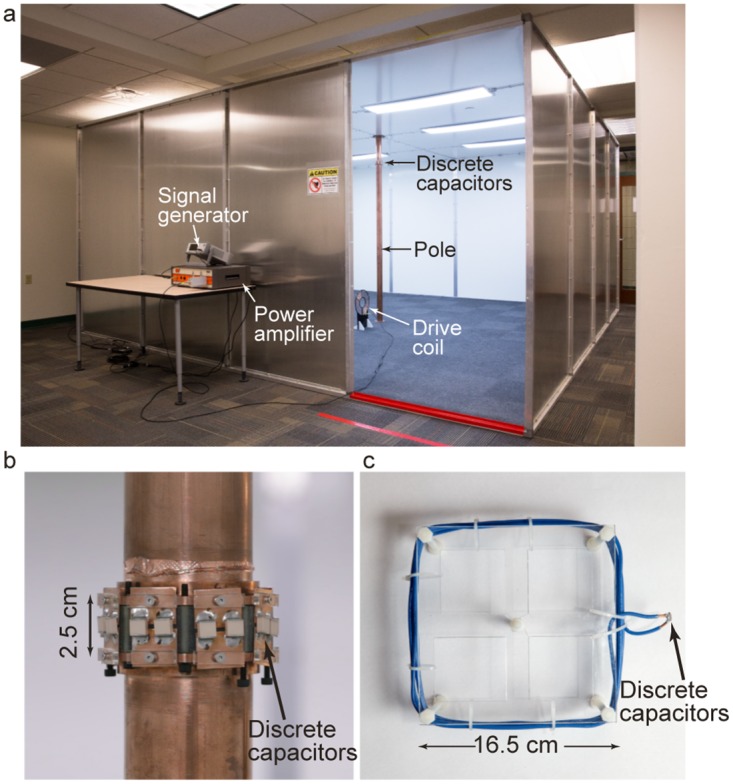
Photographs of the experimental setup. (a) Image of the QSCR wireless power room as viewed from the outside (b) Close up image of the central copper pole and discrete capacitors inserted across the gap. (c) Photo of the multi-turn, square receiver coil used to measure WPT efficiency.

The above theoretical derivation was experimentally validated using the QSCR wireless power room shown in Fig 3a. The room has dimensions 16′ × 16′ × 7.5′ (4.9 × 4.9 × 2.3 m) and the floor, ceiling, and walls are made of painted aluminum sheet metal, bolted to an aluminum frame (with gray carpet covering the floor).

The QSCR room has a central copper pole with diameter 7.2 cm, with 15 high-Q discrete capacitors totaling 7.3 pF inserted across a 2.5 cm gap in the pole (Fig 3b), producing resonance at 1.32 MHz. A 4′ × 7.5′ opening serves as a door; this missing panel had negligible effect on system performance. In order to measure WPT efficiency a 6-turn, 16.5 cm wide, square coil receiver is used as shown in Fig 3c. Finally, a 28 cm, 8-turn, spiral drive coil is used to stimulate the room (Fig 3a).

**Fig 4:**
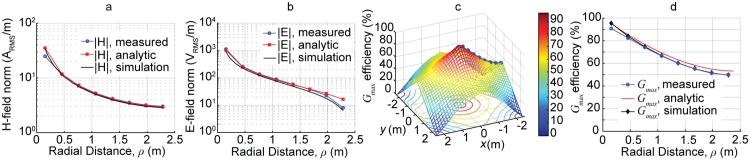
Measured and theoretical results. Measured, simulated, and analytically computed magnetic fields, (a), and electric fields, (b), when 15 W is transferred to a receiver at 50% efficiency. (c) Analytically computed WPT efficiency, *G*_*max*_ between the QSCR room and the receiver of Fig 3c. The blue dotted line shows where the data in panel (d) is taken. (d) Line-slice plot of *G*_*max*_ vs. distance from center of wireless power room.

As a first step in verifying the analytic model, 15 Watts of RF power was transmitted to the square coil receiver at an RF-to-RF efficiency of 50%. A Narda-550 high frequency broadband field meter was moved along the red line in Fig 1a, at the height of the capacitors (*z* = 2 m), which corresponds to the radial slice with the highest E-field levels. Results for the measured magnetic and electric fields in the QSCR room are shown in Fig 4a and 4b and are in good agreement with both theory and simulated results obtained from COMSOL Multiphysics commercial finite element method (FEM) software.

After confirming that the analytic model can accurately predict the electromagnetic fields in the QSCR, attention turns to the WPT efficiency. The resonant frequencies, *ω*_1,2_ of the cavity and receiver are both tuned in isolation to be 1.32 MHz. Using standard RF measurement techniques [28], the Q-factor of the QSCR room is found to be 2130 and the Q-factor of the receiver coil is 360. It is important to note that the QSCR room has a Q-factor an order of magnitude larger than can be achieved using conventional coil resonators in the same frequency range. This is due to the large amount of magnetic energy stored in the room, in addition to low ohmic losses of the wide pole and aluminum panels.

Using Eq (8), the coupling coefficient (*κ*) between the QSCR and the small square receive coil can be calculated for all positions in the room. This intermediate result, along with the measured Q-factor of the QSCR room (*Q*_1_) and square receiver coil (*Q*_2_), can be used in Eq (11) to predict wireless power transfer efficiency at any location in the room. The results are plotted in Fig 4c which shows power transfer efficiency for a horizontal 2D slice of the room. Since the magnetic field is invariant with respect to the z-height, the WPT efficiency is also invariant to the receiver’s *z*-position. A peak efficiency of 95% occurs when the receiver is placed near the pole and falls off to about 40% near the walls. This results in approximately 80% of the room’s 54 m^3^ total volume being able to deliver wireless power to a receiver at over of 40% efficiency.

These analytic results are validated experimentally using a vector network analyzer (VNA) to measure the transfer efficiency from the QSCR room to the receiver coil, as it was moved radially away from the pole. Using de-embedding techniques to account for potential losses in the matching network used in the measurement setup, allows for a direct comparison to the theoretically calculated (*G*_*max*_). The measured results are shown as the blue line in Fig 4c. A detailed comparison is shown Fig 4d which shows good agreement between measured, analytic, and simulated wireless power transfer efficiency. These results show that indeed wireless power can be delivered to nearly any location in the room. It should be noted that in this initial work, the coil receiver must be orientated radially to the pole to maximize the amount of captured magnetic flux (i.e. **n** = *a*_*ϕ*_). Receivers can gain orientation insensitivity by using 3 orthogonal coils [29, 30].

## Safety

If the vision of ubiquitous wireless power in everyday environments is to be realized, then it must be safe for the general public while delivering useful amounts of power. The IEEE and FCC have adopted two sets of safety guidelines. The first, based on direct measurement of the electric field, establishes an “action level” threshold of 614 V/m (RMS) for frequencies below 1.34 MHz, at which point further investigation and safety analysis is required. Given the good agreement between predicted and measured electric field values, Eq (7) can be used to calculate the input power level that meets this safety guideline. The second and more rigorous safety metric is Specific Absorption Rate (SAR), which is a measure of how much power is absorbed by biological tissue.

**Fig 5:**
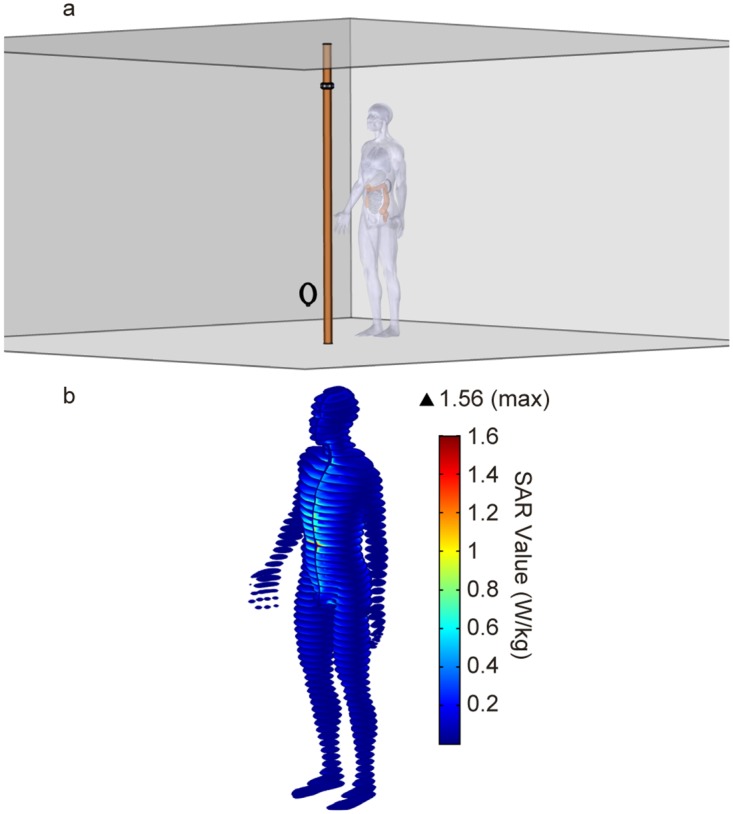
SAR simulation. (a) Setup of SAR simulation in finite element software. (b) Horizontal slices of local SAR values when the pole carries a 140 A current.

To this end, we performed a standard SAR analysis using COMSOL Multiphysics and a CAD model of an adult male body developed from full-body MRI scans [31]. The model consists of a 1.78 m (5’10”) male human body as shown in Fig 5a. Since the operating wavelength of the system is much longer than the dimensions of a human and since the field distribution is highly uniform and falls off monotonically the internal geometry of the body model can be simplified without loss of accuracy, allowing for the finite element simulation to become tractable. Once the human body model was properly meshed, the internal organs and tissues were annotated with their corresponding electromagnetic properties. The model was positioned facing the pole approximately 46 cm from the center of the room, Fig 5a.

In simulation, the current in the pole was increased until either the model’s whole body SAR value or localized SAR [10] value met the established threshold for uncontrolled exposure for the general public. This resulted in a maximum current in the pole of 140 amps and a plot of the pointwise SAR values in the human body model are shown in Fig 5b. While the peak pointwise SAR values are near the 1.6 W/kg limit. It should be noted that the 1.6 W/kg limit is defined as the average over a 1 g tissue sample and thus we are assured that the average value is indeed below the threshold. Additionally, at the same 140 amp input current, the whole body average SAR value is approximately 0.06 W/kg, while the limit is 0.08 W/kg. Thus, based on both whole body SAR values and pointwise SAR values, 140 amps is considered a conservative upper bounds on the maximum amount of current that can be safely induced in the pole.

The next step is to map the 140 Amps of current in the pole to the corresponding transmit and receive power levels. However, the magnitude of the current in the pole is dependent on loading effects. For instance under no load (e.g. 0% efficiency) it takes a smaller amount of input power to induce 140 Amps into the pole and thus meet the SAR safety limit. In contrast, under high loading conditions (e.g. 90% efficiency) more power can be injected into the QSCR room –since most of it will be delivered to the load– before the 140 Amp limit is met. Thus in order to quantify safety in terms of input power, simulations have been done at various transfer efficiency levels.

**Fig 6:**
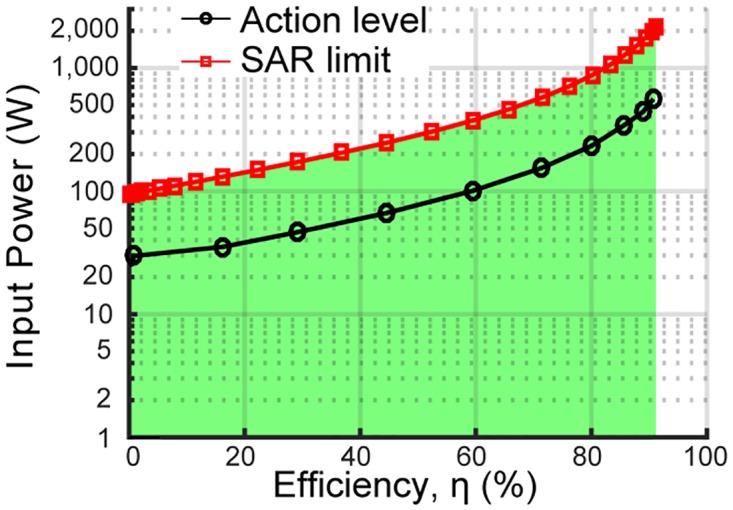
Safe input power thresholds. Maximum permissible power levels (green region) as a function of transfer efficiency. Red line shows where SAR limit is exceeded when the human body model is 46 cm away from the central pole, and the black line is the action level or where the E-field magnitude exceeds 614 V/m at 46 cm away from the pole.

The results of the safety analyses based on electric field magnitude (i.e. the “action level”) and SAR level are shown in Fig 6. These results show that it is possible to safely transmit 1.9 kilowatts of power to a receiver at 90% efficiency, which is equivalent to charging 320 USB powered devices. However, there is a dependency between the maximum permissible power level and transfer efficiency, since unused power is stored in the high Q-factor QSCR room. While standard methods such as real-time power tracking can be used to monitor the link efficiency between the room and receiver to ensure safe operation, it should be noted that even at the low end of the efficiency scale it is possible to safely transmit 100 watts of power, providing a significant amount of utility. Finally, for distances close to the pole (i.e. < 46cm) standard RF safety strategies such as intrusion detection or adding a mechanical keep-out in the form of a decorative wall can be employed.

## Discussion and Conclusion

While this article focuses on proving the underlining physics of QCSR and demonstrating safe, room scale wireless power delivery. This technique can be applied to a wide variety of usage scenarios from small charging cabinets, to midsize rooms and offices, to large-scale warehouses potentially using multiple poles.

**Fig 7:**
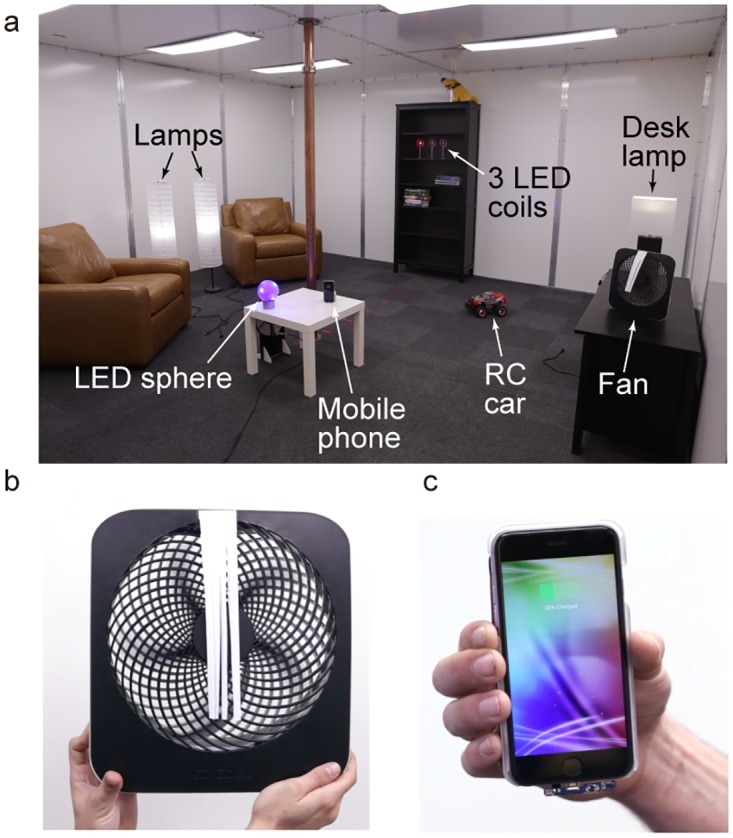
Example devices being powered in the experimental quasistatic cavity resonator room. (a) Photo showing simultaneous powering of multiple devices in a realistic living room type environment. (b) Close-up photo of a 5 W fan being powered wirelessly near the room’s wall. The receiver coil is hidden inside the casing, wound around the perimeter. (c) Close-up photo of a mobile phone being powered wirelessly within the room.

As a final demonstration of the utility of this technique Fig 7a shows the QSCR room furnished with standard bookshelves, chairs and tables. All of the labeled electronic devices have been augmented with wireless power receivers. The signal generator, power amplifier and drive coil (see Fig 3) are use to inject 15 watts of RF power into the room, which is able to simultaneously power all ten devices. Fig 7b and 7c show detailed views of the desk fan and mobile phone being powered in the room. A video demonstration of the WPT room is included in the supplementary material (see S1 Video) and provides a qualitative understanding of the volume of space that power can be delivered to, as well as the ease of use when recharging mobile devices.

One of the key benefits using in magnetic fields in the low megahertz frequency range is that they do not interact with common everyday materials. Metal objects such as phones, lamps and office furniture do not strongly couple to the QSCR and importantly do not suffer from eddy current heating, which is typical in low frequency inductive systems. Finally, the high Q-factor and sub-wavelength operation of the QSCR room permits the inclusion of windows and doors, without significantly altering system performance. In the long term we believe the requirement of metalized walls, ceilings and floors can be significantly reduced by optimizing the QSCR, and retrofitting of existing structures will be possible via modular panels or conductive paint. Ultimately, QCSR based wireless power offers a viable method for eliminating the wires and batteries that have limited many innovative solutions in the industrial, medical, and consumer electronic spaces while providing an unprecedented amount of spatial charging freedom.

## Supporting Information

S1 Appendix Room Construction. (PDF)

S2 Appendix Details on Derivation of QSCR Magnetic and Electric Fields. (PDF)

S1 Video Wireless Power Room Demo Video. Included with this article is a video showing how the wireless power QSCR room operates. Examples of wirelessly powering LEDs and other electric devices are shown and used to illustrate how power can be provided to devices anywhere in the room and with orientation insensitivity. The video also shows how wireless powering of many devices simultaneously can be accomplished in a realistic living room environment, and at field levels safe for human occupancy.
(MP4)

S1 Datalink (TXT)

S2 Dataset (Zip)
