# Supplementary material for: Quasistatic Cavity Resonance for Ubiquitous Wireless Power Transfer
Source: PLoS One. 2017 Feb 15;12(2):e0169045. doi: 10.1371/journal.pone.0169045 (PMC5310757; doi:10.1371/journal.pone.0169045)
Supplement: S1 Appendix — (PDF) [file pone.0169045.s001.pdf]

S1 Appendix: Room Construction

The QSCR wireless power room has dimensions $16' \times 16' \times 7.5'$ ($4.9 \times 4.9 \times 2.3$ m). The room was constructed using a slotted aluminum framing system to create an exoskeleton and covered with $4'$ (1.2 m) wide panels of $0.04''$ thick, 1100 series aluminum sheet metal. Although care was taken to insure good electrical contact between the panels and the framing system, no specialized construction techniques where employed beyond the use of hand tools. For aesthetic reasons the aluminum panels were painted white and the floor was covered with gray carpet tile. Ceiling lighting panels have been added (which are externally powered). Finally as can be seen in Fig. 3a of the main text, one of the wall panels was never installed and serves as an entryway.

The central pole consists of two pieces of $2.85''$ (outer diameter) hollow copper pole, which axially sandwiches a $1''$ (2.54 cm) 3D printed plastic spacer. The 3-piece assembly is bolted together with custom copper mounting flanges. The mounting flange doubles as a bracket to hold the discrete capacitors as shown in Fig. 3b of the main text. The entire pole assembly is installed vertically in the center of the room with a second set of copper mounting flanges.

In all experiments, the room was stimulated using an 8-turn spiral input coil whose outer diameter was 28 cm, Fig. 3a. By adjusting the separation between the input coil and the pole, the system could be tuned to match the 50 Ω source of either the VNA used in efficiency measurements or the 50 Ω source of the power amplifier used in the high power experiments when measuring the electric and magnetic fields, as well as powering devices.
