# Supplementary material for: Quasistatic Cavity Resonance for Ubiquitous Wireless Power Transfer
Source: PLoS One. 2017 Feb 15;12(2):e0169045. doi: 10.1371/journal.pone.0169045 (PMC5310757; doi:10.1371/journal.pone.0169045)
Supplement: S2 Appendix — (PDF) [file pone.0169045.s002.pdf]

S2 Appendix: Details on Derivation of QSCR Magnetic and Electric Fields

In the main text, the integral in Eq (3) has no closed form solution. To approximate the answer, first define the integrand of Eq (3) as the differential z-component of the magnetic vector potential:

$$dA_z = \frac{\mu_o I_o}{4\pi} \frac{\cos^2[k(\frac{h}{2} - |z'|)]}{\left[\rho^2 + (z - z')^2\right]^{\frac{1}{2}}} \tag{A.1}$$

Next, it is possible to compute the differential magnetic field from the relationship $\mathbf{dH} = \frac{1}{\mu_o} \nabla \times dA_z \mathbf{a_z}$. Evaluating this curl produces

$$\mathbf{dH} = \frac{I_o}{4\pi} \frac{\cos^2[k(\frac{h}{2} - |z'|)]\rho}{\left[\rho^2 + (z - z')^2\right]^{\frac{3}{2}}} \mathbf{a}_\phi, \tag{A.2}$$

This approach allows us to recover the magnetic field by integrating Eq (A.2):

$$\mathbf{H} = H_\phi \mathbf{a}_\phi = \frac{I_o}{4\pi} \int_{-\infty}^{\infty} \frac{\cos^2[k(\frac{h}{2} - |z'|)]\rho}{\left[\rho^2 + (z - z')^2\right]^{\frac{3}{2}}} dz' \mathbf{a}_\phi \tag{A.3}$$

A good approximation to the solution of Eq (A.3) can be made by recognizing that almost all of the area under the integrand's curve is accumulated when $z' = z$. Since z is between $\pm h$, the $\cos^2$ term is nearly constant and can be moved outside the integral in Eq (A.3)

$$H_\phi \approx \frac{I_o}{4\pi} \cos^2[k(\frac{h}{2} - |z|)] \int_{-\infty}^{\infty} \frac{\rho}{(\rho^2 + (z - z')^2)^{\frac{3}{2}}} dz' \tag{A.4}$$

Then, integration of what remains in Eq (A.4) yields the H-field due to the QSCR's central pole as presented in the main text:

$$\mathbf{H} = H_\phi \mathbf{a}_\phi \approx \frac{I_o}{2\pi} \frac{\cos^2[k(\frac{h}{2} - |z|)]}{\rho} \mathbf{a}_\phi \tag{A.5}$$

Next, attention turns to calculation of the E-field. To do this we curl the differential H-field, $dH_\phi \mathbf{a}_\phi$ of Eq (A.2) and integrate the result to obtain the E-field. Thus,

$$\mathbf{dE} = \frac{1}{j\omega_o \epsilon_o} \nabla \times \mathbf{dH} \tag{A.6}$$

where ω_o is the frequency and ϵ_o is the permittivity of free space. Then, using Eq (A.2) in (A.6) yields

$$\mathbf{dE} = -j \frac{I_o}{\pi \omega_o \epsilon_o} \left\{ \left[\frac{3}{4} \frac{\rho \cos^2[k(\frac{h}{2} - |z'|)](z)}{[\rho^2 + (z - z')^2]^{5/2}} - \frac{3}{4} \frac{\rho \cos^2[k(\frac{h}{2} - |z'|)](z')}{[\rho^2 + (z - z')^2]^{5/2}} \right] \mathbf{a}_\rho + \left[\frac{1}{2} \frac{\cos^2[k(\frac{h}{2} - |z'|)]}{[\rho^2 + (z - z')^2]^{3/2}} - \frac{3}{4} \frac{\rho^2 \cos^2[k(\frac{h}{2} - |z'|)]}{[\rho^2 + (z - z')^2]^{5/2}} \right] \mathbf{a_z} \right\} \tag{A.7}$$

Like with the integral that produced the magnetic field, each of the terms to be integrated in Eq (A.7) has a maximum when $z = z'$, which is where most of the area under the integrand's curve is accumulated when integrating. To yield a non-zero answer, however, a two term Taylor approximation around $z = z'$ can be used to replace the $\cos^2$ term in Eq (A.7), permitting integration over z'. The two-term Taylor approximation for the $\cos^2$ function in a neighborhood around $z = z'$ is

$$\cos^2[k(\tfrac{h}{2}-|z'|)] \approx \begin{cases} \cos\left[k(\frac{h}{2}-z)\right] + 2k\sin\left[k(2|z|-h)\right](z'-z), & z'>0 \\ \cos\left[k(\frac{h}{2}+z)\right] - 2k\sin\left[k(2|z|-h)\right](z'-z), & z'<0 \end{cases} \tag{A.8}$$

Using this in place of the $\cos^2$ terms in Eq (A.7) and integrating over all real z' yields the result for the E-field due to the QSCR's central pole as presented in the main text:

$$\mathbf{E} = \frac{-j\eta_o I_o}{2\pi}\sin[k(2\,|z|-h)]\left[\frac{z^3}{(\rho^2+z^2)^{3/2}\rho}\mathbf{a}_\rho + \frac{\rho^2+2z^2}{(\rho^2+z^2)^{3/2}}\mathbf{a_z}\right] \tag{A.9}$$

where η_o is the impedance of free space.
